# BRCA1 subcellular localization regulated by PI3K signaling pathway in triple-negative breast cancer MDA-MB-231 cells and hormone-sensitive T47D cells

**DOI:** 10.1515/biol-2020-0054

**Published:** 2020-07-10

**Authors:** Bin Ma, Wenjia Guo, Meihui Shan, Nan Zhang, Binlin Ma, Gang Sun

**Affiliations:** Department of Breast and Head & Neck, The Affiliated Cancer Hospital of Xinjiang Medical University, No. 789 Suzhou East Street, Urumqi 830011, Xinjiang, P. R. China; Xinjiang Uygur Autonomous Region Cancer Research Institute, Urumqi 830011, Xinjiang, P. R. China

**Keywords:** breast cancer, BRCA1 subcellular localization, PI3K signaling pathway

## Abstract

This study is to investigate the effect of the PI3K/Akt signaling pathway on the regulation of BRCA1 subcellular localization in triple-negative breast cancer (TNBC) MDA-MB-231 cells and hormone-sensitive T47D cells. We found that heregulin-activated T47D cells showed more nuclear localization of BRCA1, but BRCA1 nuclear localization decreased after the inhibition of the PI3K signaling pathway. In MDA-MB-231 cells, activation or inhibition of the PI3K signaling pathway did not significantly affect cell apoptosis and BRCA1 nuclear translocation (*P* > 0.05). However, in T47D cells, the activation of the PI3K pathway significantly increased cell apoptosis (*P* < 0.05). In the heregulin-activated MDA-MB-231 and T47D cells, the phosphorylation of Akt and BRCA1 was significantly increased (*P* < 0.05), while that was significantly reduced after PI3K pathway inhibition (*P* < 0.05). The changing trends of the mRNA levels of *Akt* and *BRCA1* in MDA-MB-231 and T47D cells after PI3K pathway activation or inhibition were consistent with the trends of their proteins. In both MDA-MB-231 and T47D cells, BRCA1 phosphorylation is regulated by the PI3K signaling pathway, but the nuclear localization of BRCA1 is different in these two cell lines. Moreover, the apoptosis rates of these two cell lines are different.

## 1 Introduction

Breast cancer is one of the most common malignant tumors among women worldwide, which seriously threatens women’s health [1]. However, the etiology and pathogenesis of breast cancer have not yet been fully elucidated. Breast cancer susceptibility gene-1 (BRCA1) has been recognized as one of the risk factors for breast cancer occurrence and development [2]. BRCA1 is an essential protein at the G2/M checkpoint, which can inhibit the cell cycle progression, regulate the DNA damage repair [3], inhibit cell proliferation, and induce apoptosis [4,5,6]. The BRCA1 gene mutations can lead to reduced BRCA1 protein expression and loss of function [7]. Meanwhile, the subcellular localization of BRCA1 also has important effects on its function. The decreased BRCA1 expression and the subcellular localization abnormalities have been observed in triple-negative breast cancer (TNBC) [8,9]. BRCA1 is mainly located in the nucleus of normal breast tissue [10]. A large number of studies have shown that after being regulated by different signals, BRCA1 can be translocated from the nucleus to the cytoplasm, which can affect its function [11,12].

The pathogenesis of breast cancer is regulated by multiple signaling pathways. Many studies have shown that the Notch signaling pathway [13,14], Wnt signaling pathway [15,16], NF-κb pathway [17], JAK-STAT signaling pathway [18], and PI3K/AKT signaling pathway [19] are all related to the occurrence and development of breast cancer. Among these signaling pathways, the PI3K/AKT signaling pathway plays a leading role. More than 70% of breast cancer cases have PIK3CA gene mutations, and the mutated PIK3CA gene can cause the activation of the PI3K/AKT pathway, which subsequently leads to rapid proliferation and invasion of breast cancer cells, probably ending up with metastasis [20]. Meanwhile, heregulin (HRG) can promote the BRCA1 phosphorylation [21] and nuclear localization [22] through activating AKT.

Our previous study [23] found that the BRCA1 expression was decreased in breast cancer tissues, and its subcellular localization changed from the nucleus to cytoplasm. The BRCA1-associated RING domain protein 1 gene, which is closely related to BRCA1, also showed subcellular localization changes. Previous studies [21,22] mainly focused on hormone-sensitive T47D breast cancer cell lines (ER and PR positive). TNBC has no receptor markers like other types of breast cancer [24] and has a poor prognosis [25,26]. There are no controlled studies concerning the changes in BRCA1 subcellular localization and function in different breast cancer types. Additionally, whether BRCA1 subcellular localization and function can be regulated by the PI3K/PTEN/AKT signaling pathway in TNBC is still unknown. This study investigated the effect of PI3K/PTEN/AKT signaling pathway on the subcellular localization of BRCA1 in the TNBC MDA-MB-231 and hormone-sensitive T47D cell lines.

## 2 Materials and methods

### 2.1 Cell lines

T47D (hormone-sensitive) and MDA-MB-231 (TNBC) cell lines were purchased from Shanghai Genechem Co., Ltd (Shanghai, China). Both cell lines were cultured in DMEM supplemented with 10% fetal bovine serum and 1% penicillin–streptomycin at 37°C under a humidified atmosphere containing 5% CO_2_.

### 2.2 Lentiviral transfection

According to the target gene sequence of AKT1 (accession number NM_001014432), three RNA interference target sequences were designed and synthesized by Shanghai Genechem Co., Ltd (Shanghai, China), namely, ATCGCTTCTTTGCCGGTAT, ACAAGGACGGGCACATTAA, and TCCTCAAGAAGGAAGTCAT. The lentiviral particles were constructed using the GV112 lentiviral vector, while the sequence of TTCTCCGAACGTGTCACGT was used to construct an unrelated sequence vector.

Cells were seeded onto 96-well plates at a concentration of 4 × 10^3^ cells/well. After 24 h of incubation, the culture medium was replaced with fresh medium containing 5 μg/mL polybrene. Lentivirus suspension at a different viral multiplicity of infection (MOI = 0, 5, 10, 20, 40) was added, and fresh medium was changed after 16 h. Puromycin was used for screening. The optimal concentration of puromycin for MDA-MB-231 cells and T47D cells was determined at 2 μg/mL and 4 μg/mL, respectively. The optimal MOI of MDA-MB-231 cells and T47D cells was 5 and 10, respectively. The successful transfection of shRNA was detected by Western blot.

### 2.3 Cell treatment and grouping

After seeding into 96-well plates, the cells were divided into different groups and treated as follows: Control group, cells were incubated with complete DMEM; PI3K activation group (HRG group), cells were treated with 30 ng/mL HRG for 24 h; PI3K inhibition group (HRG + LY294002 group), cells were treated with 30 ng/mL HRG for 24 h and then 25 μM LY294002 for 48 h; Akt silence group (HRG + ShRNA group), cells were treated with 30 ng/mL HRG for 24 h and then Akt was knocked down with lentivirus-mediated Akt-specific small interfering RNA for 16 h, and incubated for another 48 h.

### 2.4 Immunofluorescence

MDA-MB-231 and T47D cell suspensions were adjusted to 4 × 10^4^/mL, and 100 µL of the cells were seeded to 96-well plate. After 24 h of incubation, cells were fixed. The rabbit anti-BRCA1 primary antibody (cat. #bs-0803R, Bioss Co., Beijing, China) was added and incubated for 20 min at room temperature. Then, goat anti-rabbit IgG/FITC antibody (cat. #bs-0295G-FITC, Bioss Co., Beijing, China) and Hoechst 33342 (5 μg/mL) (cat. #B8040, Solarbio Co., Beijing, China) were added and incubated at room temperature for 15 min. Finally, the cells were observed under a fluorescence microscope (Axio Observer A1, Zeiss Co., Germany).

### 2.5 Flow cytometry

The cells were suspended in 100 μL PBS at a concentration of over 1 × 10^7^ cells/mL. Annexin V-FITC/PI cell apoptosis kit (Solarbio Co., Beijing, China) was used to stain the cells for 20 min at room temperature in the dark. After washing and resuspension, the cell apoptosis was detected with a flow cytometer (Bricyte E6, Mindray Bio-Medical Electronics Co., Ltd, Shenzhen, China).

### 2.6 Western blot

The total protein was extracted, and the protein concentration was determined using the BCA method. The protein samples were subjected to SDS-PAGE and then transferred onto PVDF membranes. The membrane was blocked with skim milk and incubated with rabbit anti-BRCA1 primary antibody, rabbit anti-phospho-BRCA1 (Ser1524) antibody (cat. #9009, Cell Signaling Technology, Inc., Danvers, MA, US), rabbit anti-Akt3 (E2B6R) mAb (cat. #14293, Cell Signaling Technology, Inc., Danvers, MA, US), rabbit anti-phospho-Akt (Thr450) (D5G4) mAb (cat. #12178, Cell Signaling Technology, Inc., Danvers, MA, US), and GAPDH antibody overnight at 4°C. After washing, the anti-rabbit IgG HRP-linked secondary antibody (cat. #7074, Cell Signaling Technology, Inc., Danvers, MA, US) (1:2,000) was added and incubated at 37°C for 1 h in the dark. GAPDH was used as an internal control. The membrane was developed with ECL. The protein expression was calculated as follows: relative expression = gray value of the target protein/gray value of GAPDH.

### 2.7 RT-PCR

Total RNA was extracted with TRIzol agent, and cDNA was synthesized using a PrimeScript™ RT reagent Kit (Takara Co., Japan). The target gene was amplified by PCR at the following condition: pre-denaturation at 94°C for 1 min, followed by 40 cycles of denaturation at 94°C for 10 s, annealing, and extension at 60°C for 34 s. The PCR product was subjected to 2% agarose gel electrophoresis and visualized. The image was analyzed by Image J2x. GAPDH was used as an internal reference. The relative mRNA level was calculated by the 2^−ΔΔCt^ method. The primer sequence was listed in Table 1.

**Table 1:** The sequence of primers used in this study

Gene	Sequence
BRCA1	Forward: 5′-TTTCCTGTGGTTGGTCAG-3′
	Reverse: 5′-TGAGTCCAGTTTCGTTGC-3′
Akt	Forward: 5′-CAAGAAGGAAGTCATCGTGG-3′
	Reverse: 5′-TCGTGGGTCTGGAAAGAGT-3′
GAPDH	Forward: 5′-CTTTGGTATCGTGGAAGGA-3′
	Reverse: 5′-AGGGATGATGTTCTGGAGAG-3′

### 2.8 Statistical analysis

The statistical analysis was performed with the statistical software SPSS 20.0 and GraphPad Prism 5.0. The measurement data were expressed as mean ± standard deviation (SD). Single-factor analysis of variance (ANOVA) was used for the comparison between multiple groups, and the LSD method was used for comparison between two groups. A *P* < 0.05 was considered statistically significant.

## 3 Results

### 3.1 The shRNA is successfully transfected into the MDA-MB-231 and T47D cells

**Figure 1:**
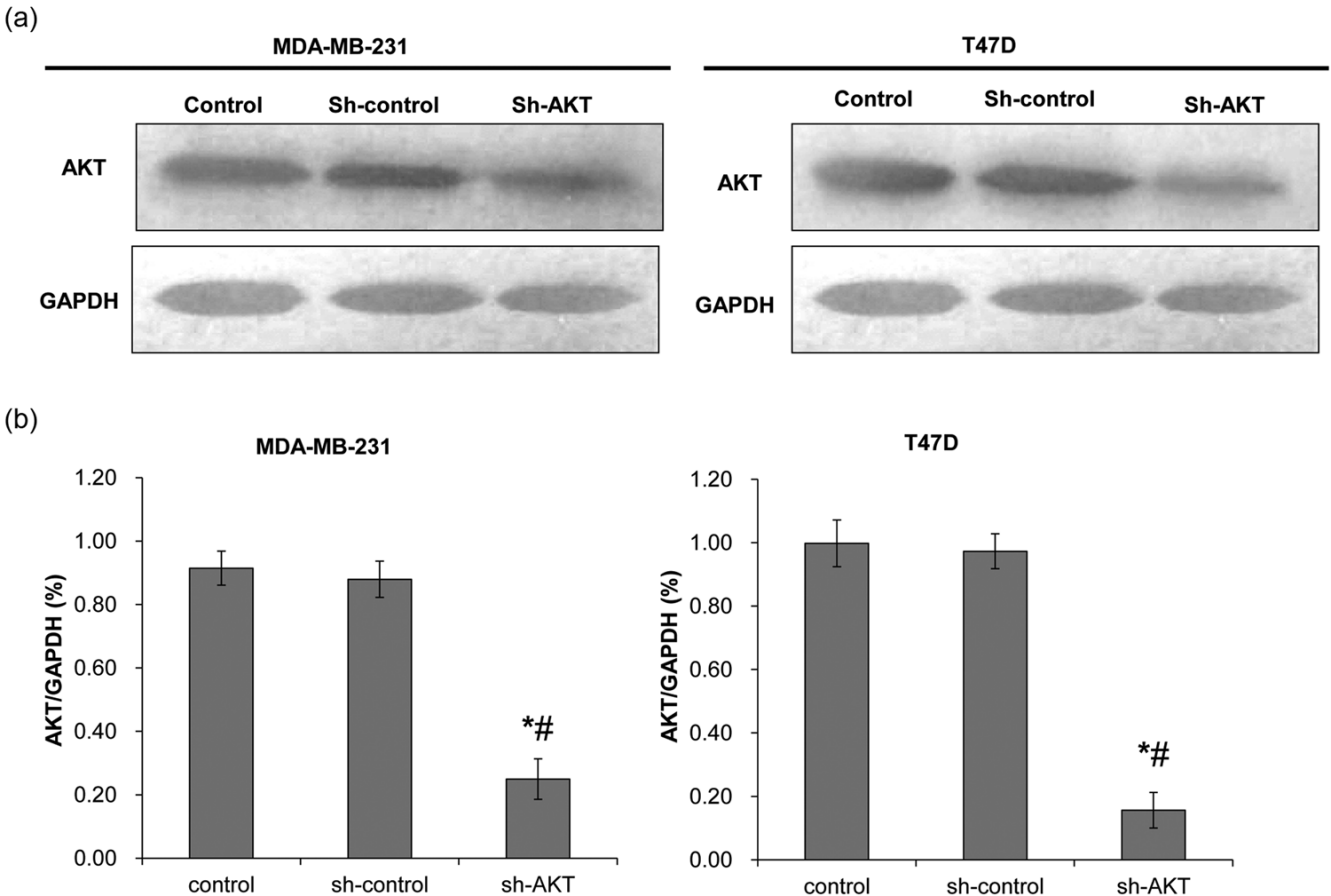
Determination of lentiviral transfection efficiency. Western blot was used to detect AKT protein expression after shRNA transfection. The control group was not transfected with shRNA; the sh-control group was transfected with void vector; and the sh-AKT group was transfected with shRNA of AKT. (a) Representative Western blot results. (b) The quantified expression of AKT. Left: MDA-MB-231 cells; right: T47D cells. *P < 0.05, compared with the control group; ^#^ *P* < 0.05, compared with the sh-control group.

The Western blot was used to detect the expression of AKT after transfection (Figure 1a and b). There was no difference in the expression of AKT between the sh-control and control groups in both MDA-MB-231 and T47D cells (*P* > 0.05). The expression of AKT in the sh-AKT group was significantly lower than that in the control and sh-control groups (*P* < 0.05). This indicates that shRNA has been successfully transfected into the MDA-MB-231 and T47D cells.

### 3.2 Regulation of BRCA1 subcellular localization by the key molecules of the PI3K signaling pathway

**Figure 2:**
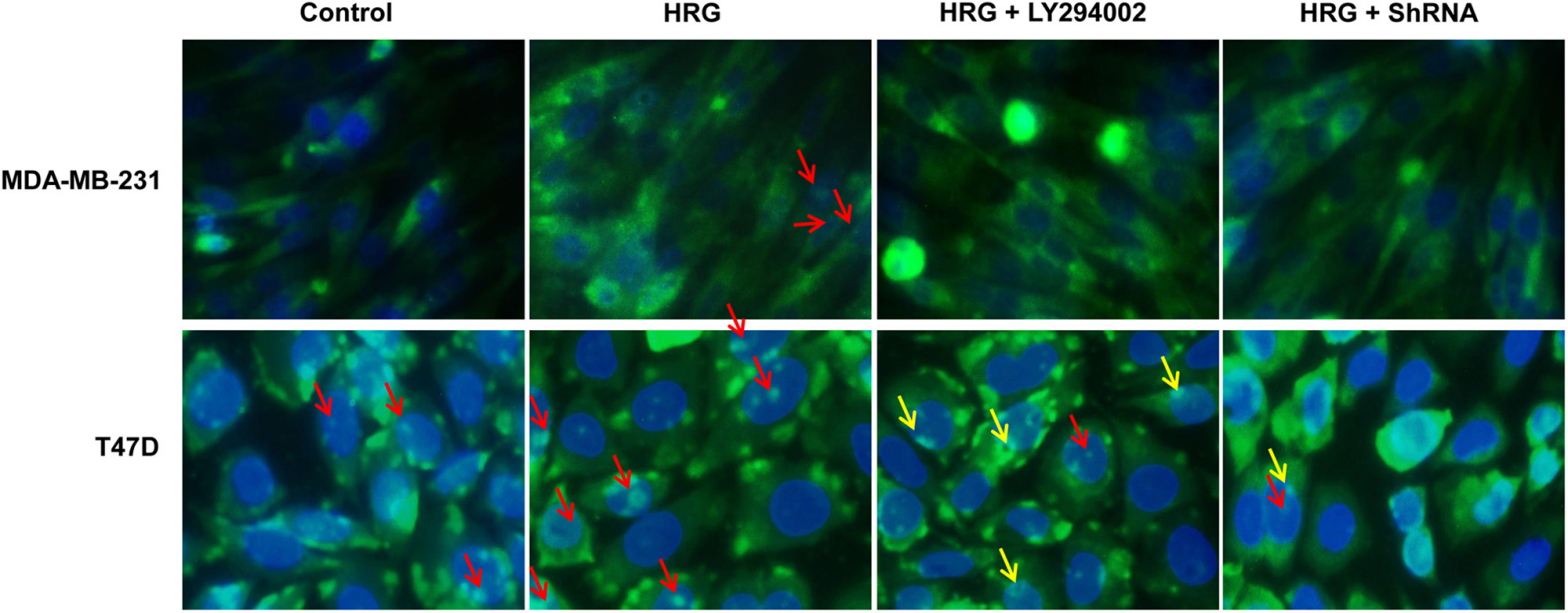
The subcellular localization of BRCA1 was observed under a fluorescence microscope (400×). The Control group was untreated. The HRG group was treated with 30 ng/mL HRG for 24 h to activate the PI3K signaling pathway. The HRG + LY294002 group was treated with 30 ng/mL HRG for 24 h to activate the PI3K signaling pathway and then treated with 25 μM LY294002 for 48 h to inhibit the PI3K pathway. The HRG + ShRNA group was treated with 30 ng/mL HRG for 24 h to activate the PI3K signaling pathway and then transfected with shRNA to silence Akt. The red arrows indicate the localization of BRCA1 protein in cell nuclei, while the yellow arrows indicate that on the edge of nuclear membranes.

To determine the subcellular localization of BRCA1, immunofluorescence was performed. As shown in Figure 2, in MDA-MB-231 cells, BRCA1 was mainly located in the cytoplasm, while only the HRG group had a very small number of BRCA1 located in the nucleus, while the other groups of cells had no obvious BRCA1 in the nucleus. In T47D cells, a small amount of BRCA1 was located in the nucleus in the Control group. In the HRG group, BRCA1 was mainly located in the nucleus. In the HRG + LY294002 group, a small amount of BRCA1 was located in the nucleus, while the remaining BRCA1 was mainly located on the edge of nuclear membranes. In the HRG + ShRNA group, BRCA1 was mainly located in the cytoplasm, and only a small part of BRCA1 was located in the nucleus (Figure 2). These results indicate that the nuclear localization of BRCA1 in both MDA-MB-231 cells and T47D cells is increased after PI3K activation, while decreased after PI3K inhibition, especially after the silencing of Akt. Moreover, the nuclear localization of BRCA1 in T47D breast cancer cells is higher than that in the MDA-MB-231 cells.

### 3.3 The effect of the PI3K signaling pathway on the apoptosis of different types of breast cancer cells

**Figure 3:**
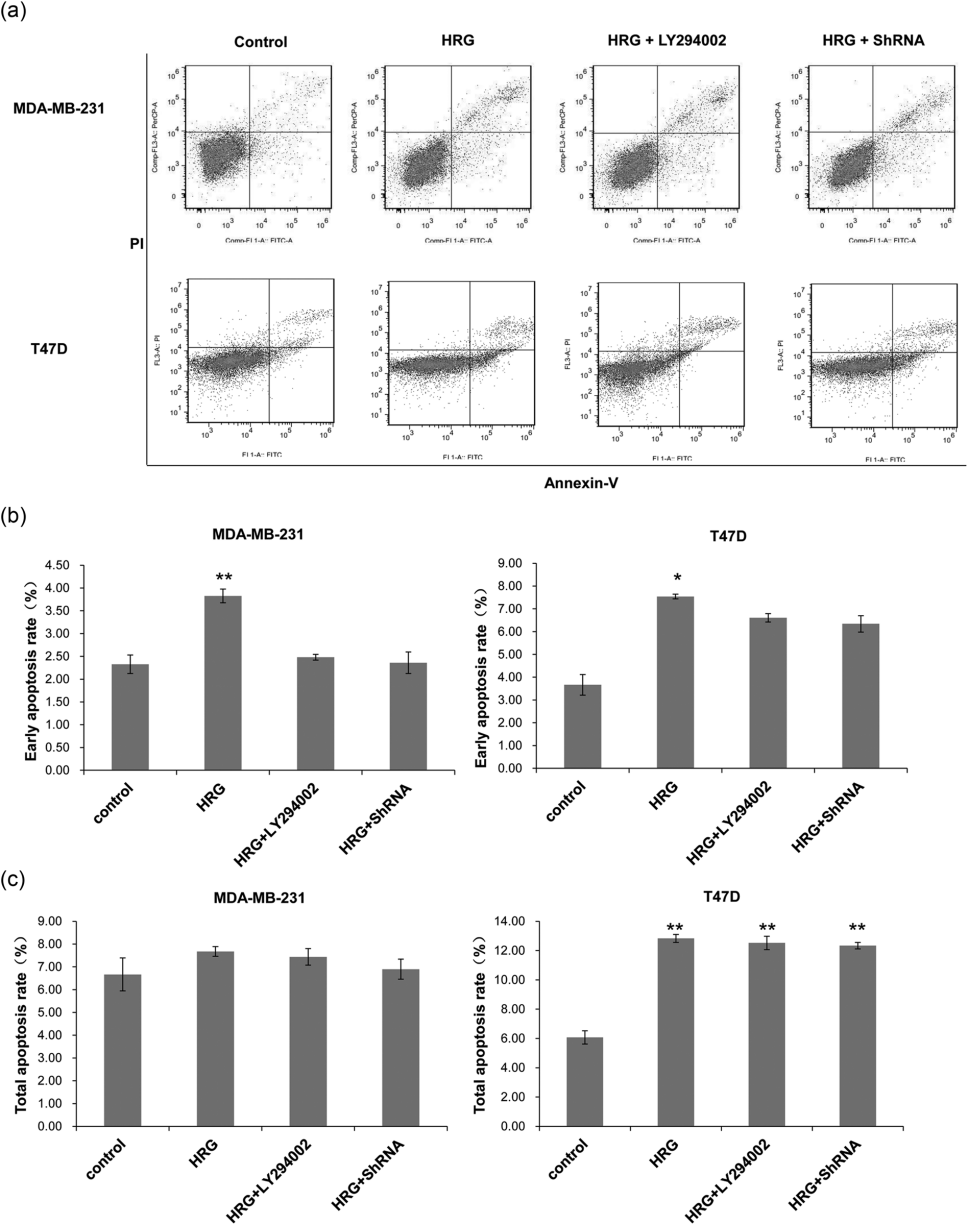
The cell apoptosis rate of different groups detected by flow cytometry. Cell treatment and grouping were described above. (a) Representative flow cytometry results. (b) The early cell apoptosis rates and (c) the total apoptosis rates of MDA-MB-231 and T47D cells. All experiments were performed at least three times. *P < 0.05, **P < 0.01, compared with the control group.

To detect cell apoptosis, flow cytometry was performed. The representative flow cytometry results are shown in Figure 3a. The early and total apoptosis rate was calculated, respectively. As shown in Figure 3b, for both MDA-MB-231 and T47D cells, the early apoptosis rates in the HRG group were significantly higher than those in the other groups (*P* < 0.05), whereas there was no significant difference between the other groups (*P* > 0.05). In MDA-MB-231 cells, the percentage of total apoptotic cells was not significantly different among all the groups (*P* > 0.05) (Figure 3c). In T47D cells, the apoptosis rate of the Control group was significantly lower than that in other groups (*P* < 0.01). However, there was no significant difference among the HRG group, HRG + LY294002 group, and HRG + ShRNA group (*P* > 0.05) (Figure 3c). This indicates that the activation of the PI3K signaling pathway can significantly increase the early apoptosis rate of cells, while the activation or inhibition of the PI3K signaling pathway has no effect on the total apoptosis rate of MDA-MB-231 cells, but can significantly increase that of the T47D cells.

### 3.4 The phosphorylation and mRNA expressions of Akt and BRCA1 in breast cancer cells

**Figure 4:**
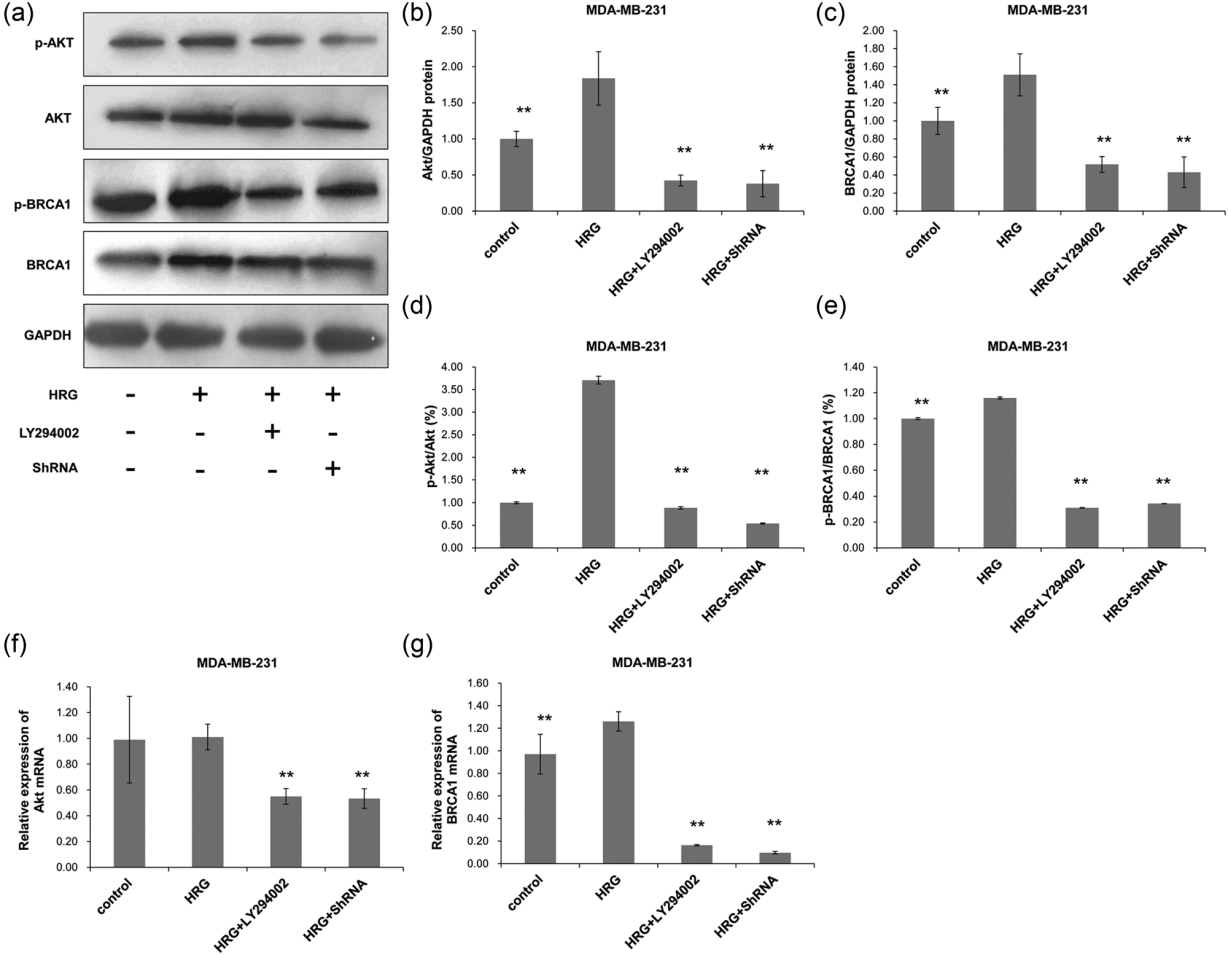
The protein and mRNA expression of Akt and BRCA1 in MDA-MB-231 cells. Cell treatment and grouping were described above. The protein and mRNA expressions were detected with Western blot and RT-PCR, respectively. (a) The protein expressions of Akt and BRCA1 and their phosphorylated counterparts. The protein expressions of (b) Akt and (c) BRCA1 were quantified in relative to that of GAPDH. (d) The percentage of p-Akt in the total Akt protein. (e) The percentage of p-BRCA1 in the total BRCA1 protein. (f) The relative expression of *Akt* mRNA and (g) the relative expression of *BRCA1* mRNA were also detected. All experiments were performed at least three times. *P < 0.05, compared with the control group; ^#^ *P* < 0.05, compared with the HRG group.

The protein levels of Akt and BRCA1 and their phosphorylation in MDA-MB-231 cells were detected with Western blot. After PI3K activation by HRG, the phosphorylation of Akt and BRCA1 increased significantly in MDA-MB-231 cells (*P* < 0.05) compared with the Control group and decreased significantly after the inhibition of PI3K pathway or silencing of Akt (*P* < 0.01) (Figure 4a–e). There was no significant difference in the phosphorylation of Akt and BRCA1 between the HRG + LY294002 and the HRG + ShRNA groups (*P* > 0.05). This suggests that HRG can promote the phosphorylation of BRCA1 by increasing the phosphorylation of Akt in the PI3K/Akt signaling pathway.

The mRNA expression levels of *BRCA1* and *AKT* in MDA-MB-231 were detected by RT-PCR. The results showed that the expression of *Akt* mRNA (Figure 4f) in MDA-MB-231 cells had no significant changes after the activation of PI3K (*P* > 0.05) when compared with that in the Control group, but the expression of *BRCA1* mRNA significantly increased (*P* < 0.05) (Figure 4g). Both *Akt* and *BRCA1* expressions significantly decreased after the PI3K pathway was inhibited or silenced (*P* < 0.05) (Figure 4f and g). This indicates that the phosphorylation and mRNA changing trends of Akt and BRCA1 in the TNBC MDA-MB-231 cells are similar after activation or inhibition of the PI3K signaling pathway.

**Figure 5:**
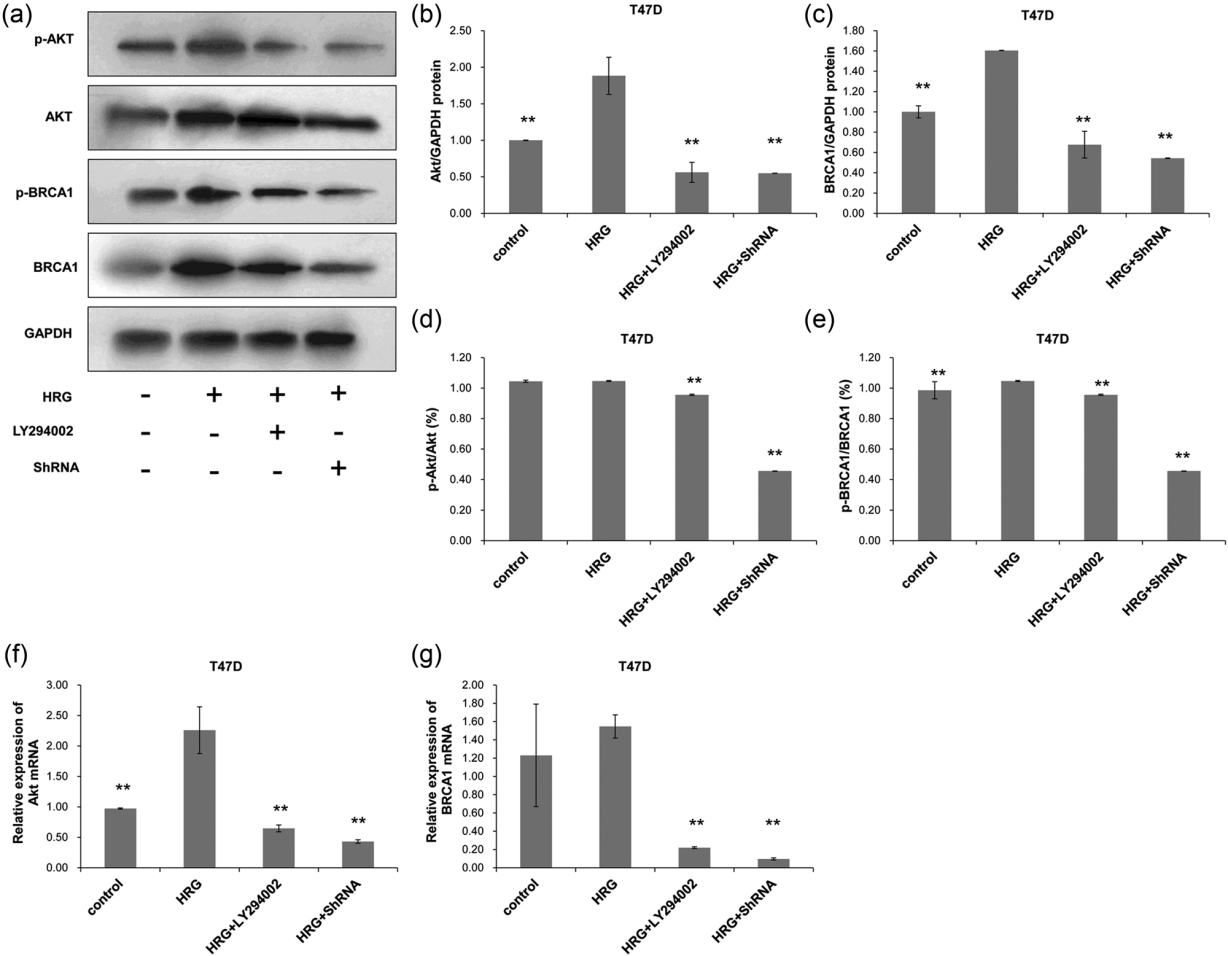
The protein and mRNA expression of Akt and BRCA1 in T47D cells. Cell treatment and grouping were described above. The protein and mRNA expressions were detected with Western blot and RT-PCR, respectively. (a) The protein expressions of Akt and BRCA1 and their phosphorylated counterparts. The protein expressions of (b) Akt and (c) BRCA1 were quantified in relative to that of GAPDH. (d) The percentage of p-Akt in the total Akt protein. (e) The percentage of p-BRCA1 in the total BRCA1 protein. (f) The relative expression of *Akt* mRNA and (g) the relative expression of *BRCA1* mRNA were also detected. All experiments were performed at least three times. *P < 0.05, compared with the control group; ^#^ *P* < 0.05, compared with the HRG group.

Compared with the Control group, the phosphorylation of Akt (Figure 5a, b and d) had no significant change when PI3K was activated in T47D cells (*P* > 0.05), but the phosphorylation of BRCA1 (Figure 5a, c and e) increased significantly (*P* < 0.05). The silencing of Akt by shRNA decreased the phosphorylation of BRCA1 significantly (*P* < 0.05) (Figure 5e) when compared with the HRG + LY294002 group. The mRNA expressions of *Akt* (Figure 5f) and *BRCA1* (Figure 5g) both significantly increased in the HRG group when PI3K pathway was activated, whereas both significantly decreased after the PI3K pathway was inhibited or silenced in T47D cells of the HRG + LY294002 group and the HRG + ShRNA group (*P* < 0.05). This indicates that the phosphorylation and mRNA changing trends of Akt and BRCA1 in the hormone-sensitive T47D cells are similar after activation or inhibition of the PI3K signaling pathway. The above results suggest that the phosphorylation of BRCA1 in MDA-MB-231 and T47D cells are both regulated by the PI3K pathway.

## 4 Discussion

BRCA1 is a tumor suppressor gene that is closely related to breast cancer, ovarian cancer, and other hormone-related cancers [27] and plays a negative regulatory role in tumor growth. It also plays an important role in regulating cell damage repair, cell cycle, and cell apoptosis. However, there are still some controversies concerning the investigation of breast cancer treatment based on BRCA1 mutations [28]. The PI3K signaling pathway is a common signaling pathway mainly involved in biological behaviors such as substance metabolism regulation, cell proliferation, and survival [29,30,31]. The abnormal activation of the PI3K signaling pathway is associated with many cancers such as liver cancer, colon cancer, bladder cancer, and breast cancer [32]. PI3K signaling pathway is one of the main signaling pathways in breast cancer. Altiok et al. [21] showed that the PI3K signaling pathway was involved in phosphorylation, nuclear transportation, and subcellular localization of BRCA1. In this study, it was found that the activation of the PI3K signaling pathway could promote the expression and phosphorylation of BRCA1 and markedly increased its nuclear localization in T47D cells. On the contrary, after the PI3K signaling pathway was inhibited, BRCA1 nuclear localization was significantly reduced in T47D cells, but no obvious changes in nuclear localization were observed in MDA-MB-231 cells. Further experimentation is needed to verify the present results. These results demonstrate that the activation of the PI3K signaling pathway in T47D cells can promote BRCA1 phosphorylation and increase its nuclear localization, which is consistent with previous studies [33,34]. However, the phosphorylation of BRCA1 was increased in MDA-MB-231, but its BRCA1 nuclear localization was not increased. Therefore, whether there are other mechanisms regulating BRCA1 nuclear translocation in MDA-MB-231 cells needs to be further studied. A possible reason may be that the PI3K signaling pathway is activated by extracellular or intracellular abnormal changes, and the expression of the downstream molecules in cells is changed through a wide range of external and internal stimulation [35,36].

The PI3K signaling pathway plays an anti-apoptotic and pro-proliferative role in cells [31,32]. Activated Akt can downregulate the transcription of pro-apoptotic genes, reduce cell apoptosis, and promote cell survival [37]. Akt can also activate a variety of downstream molecules such as mTOR, Bad, ikB, caspase-9, MMP-9, COX-2, etc., thereby stimulating cell differentiation and proliferation and inhibiting apoptosis [38,39,40]. This study found that PI3K inhibition or Akt knockdown significantly increased cell apoptosis in T47D cells, which is consistent with a previous study [41]. However, the apoptotic rate of T47D cells significantly increased after the activation of PI3K, which is possibly due to the tumor suppressor function of phosphorylated BRCA1, leading to an increased apoptosis rate of cells. In MDA-MB-231 cells, there was no significant change in the apoptosis rate, which may be due to the failure of the nuclear translocation of the BRCA1 protein. Akt is the main target gene of PI3K, and its silencing can cause a decrease of BRCA1 protein in different types of breast cancer cells [21,22,42], suggesting that PI3K pathway can regulate the expression level of BRCA1. In the present study, the expression levels of BRCA1 and Akt showed a consistent changing trend.

## 5 Conclusions

This study found that the regulation of PI3K signaling pathway on BRCA1 subcellular localization was different between MDA-MB-231 (TNBC type) and T47D (hormone-sensitive type) cells. After the activation or inhibition of the PI3K pathway, the changes in nuclear localization of BRCA1, and the rate of apoptosis in T47D cells were more significant when compared with those in MDA-MB-231 cells. The underlying mechanism needs to be further studied. This study provides some clues in the treatment of different types of breast cancer.
