# Erratum to “BRCA1 subcellular localization regulated by PI3K signaling pathway in triple-negative breast cancer MDA-MB-231 cells and hormone-sensitive T47D cells”

**DOI:** 10.1515/biol-2022-0810

**Published:** 2023-12-16

**Authors:** Bin Ma, Wenjia Guo, Meihui Shan, Nan Zhang, Binlin Ma, Gang Sun

**Affiliations:** Department of Breast and Head & Neck, The Affiliated Cancer Hospital of Xinjiang Medical University, No. 789 Suzhou East Street, Urumqi 830011, Xinjiang, P. R. China; Xinjiang Uygur Autonomous Region Cancer Research Institute, Urumqi 830011, Xinjiang, P. R. China

In the published manuscript, BRCA1 subcellular localization regulated by PI3K signaling pathway in triple-negative breast cancer MDA-MB-231 cells and hormone-sensitive T47D cells. Bin Ma, Wenjia Guo, Meihui Shan, Nan Zhang, Binlin Ma and Gang Sun, Open Life Sci. 2020 Jul 10;15(1):501–510. doi: 10.1515/biol-2020-0054, the authors have found an unintentional error made during preparation of Figure 1a.

**Figure 1:**
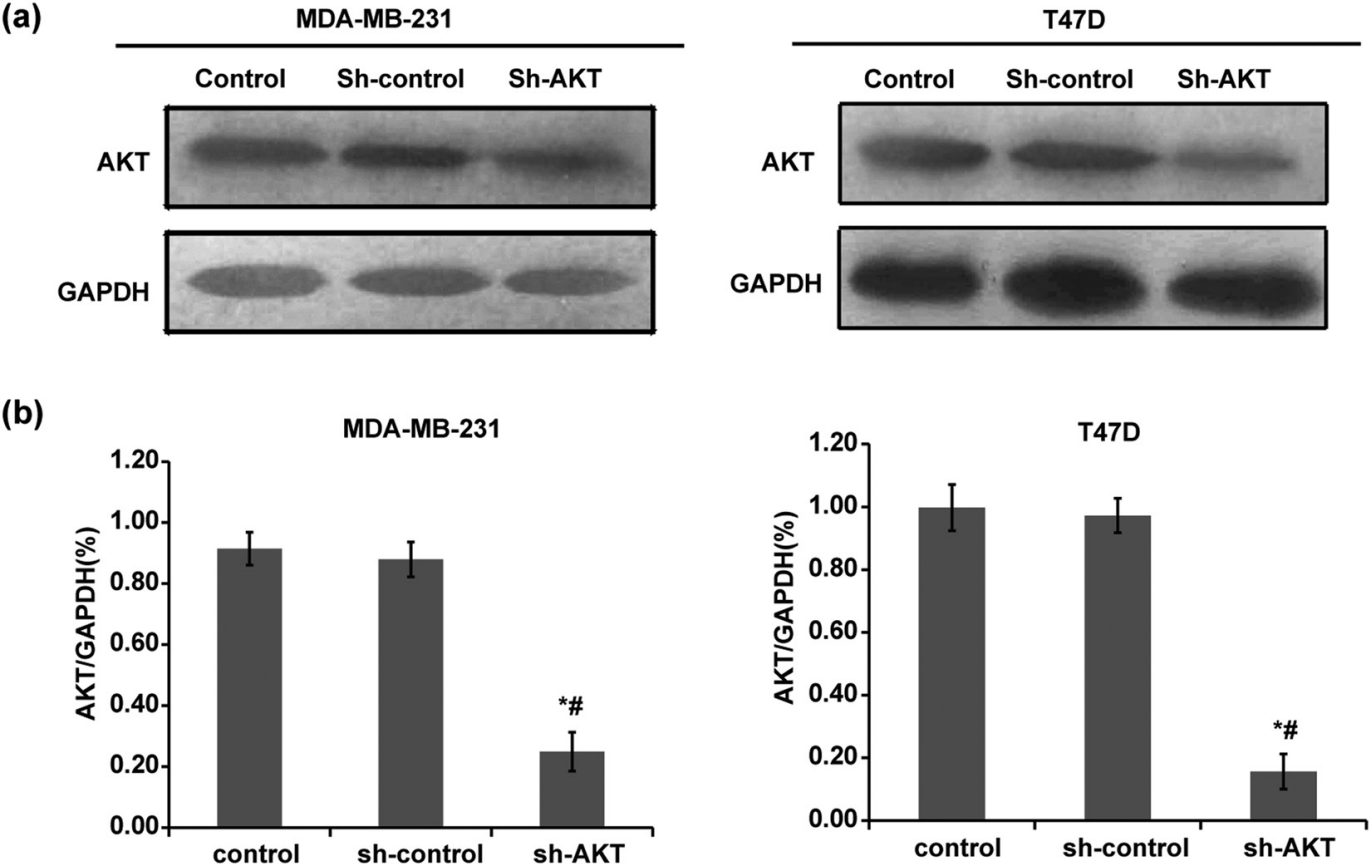
Determination of lentiviral transfection efficiency. Western blot was used to detect AKT protein expression after shRNA transfection. The control group was not transfected with shRNA; the sh-control group was transfected with void vector; and the sh-AKT group was transfected with shRNA of AKT. (a) Representative Western blot results. (b) The quantified expression of AKT. Left: MDA-MB-231 cells; right: T47D cells. *P < 0.05, compared with the control group; ^#^ *P* < 0.05, compared with the sh-control group.

The corresponding author states as follows: I just realized that in the version of the article initially published, there was an error in Figure 1a. Due to carelessness, the GAPDH for MDA-MB-231 and T47D was erroneously reused. The other elements of the figure remain the same, and the interpretation of the results remains unchanged.

The revised Figure 1 has been provided.

The authors admit the error and claim that this is an unintentional error that has nothing to do with academic misconduct and does not influence the conclusion of the publication. The authors apologize to the editor, the staffs of the journal and the journal readers for the mistake and any inconvenience it caused.

